# Special nuclear layer contacts between starburst amacrine cells in the mouse retina

**DOI:** 10.3389/fopht.2023.1129463

**Published:** 2023-03-24

**Authors:** Shang Mu, Nicholas L. Turner, William M. Silversmith, Chris S. Jordan, Nico Kemnitz, Marissa Sorek, Celia David, Devon L. Jones, Doug Bland, Merlin Moore, Amy Robinson Sterling, H. Sebastian Seung

**Affiliations:** ^1^ Princeton Neuroscience Institute, Princeton University, Princeton, NJ, United States; ^2^ Computer Science Department, Princeton University, Princeton, NJ, United States

**Keywords:** starburst amacrine cells (SACs), electron microscopy, retina, 3D reconstruction, perisomatic contact

## Abstract

Starburst amacrine cells are a prominent neuron type in the mammalian retina that has been well-studied for its role in direction-selective information processing. One specific property of these cells is that their dendrites tightly stratify at specific depths within the inner plexiform layer (IPL), which, together with their unique expression of choline acetyltransferase (ChAT), has made them the most common depth marker for studying other retinal neurons in the IPL. This stratifying property makes it unexpected that they could routinely have dendrites reaching into the nuclear layer or that they could have somatic contact specializations, which is exactly what we have found in this study. Specifically, an electron microscopic image volume of sufficient size from a mouse retina provided us with the opportunity to anatomically observe both microscopic details and collective patterns, and our detailed cell reconstructions revealed interesting cell-cell contacts between starburst amacrine neurons. The contact characteristics differ between the respective On and Off starburst amacrine subpopulations, but both occur within the soma layers, as opposed to their regular contact laminae within the inner plexiform layer.

## Introduction

An abundant and well-studied cell type in the mammalian retina is the starburst amacrine cell (SAC). These are named for the characteristic starburst shape of their dendritic trees (1, 2). They exist in two homologous subgroups: On SACs have their cell bodies in the ganglion cell layer and primarily respond to bright light stimuli, whereas Off SACs have their cell bodies in the inner nuclear layer and are better associated with the transition of stimuli from light to dark. Initially identified as the acetylcholine-synthesizing cells in the retina (2–4), these cells have well-known identifying molecular labels (5, 6), and their dendrites are narrowly stratified (1, 7), making SACs the most commonly used location-referencing landmarks for studying cells in the context of the inner plexiform layer (IPL) of the retina (8, 9).

By densely reconstructing and examining 199 starburst cells from an electron-microscopically (EM) imaged volume of a mouse retinal patch (10) in a similar manner to that previously reported (11–13), we discovered interesting contact patterns.

Specifically, these contacts are located on the cell bodies of other starburst cells from the same On or Off subgroup. This is surprising because SACs have their dendrites tightly stratified in the IPL and normally make synapses and contacts with each other and with other types of cells *via* their dendrites within the IPL, as opposed to in the nuclear layers (14–16). We found morphological characteristics specific to each of the two respective subpopulations.

Previous studies on SAC dendritic connections have mostly focused on specific cell examples in relation to their neighbors and made localized observations on specific dendritic regions of interest (e.g., 7, 10, 17, 18). Population-level observations, such as those on spatial somatic arrangements, have largely compared wild-type to transgenic strains and have not or were unable to comprehensively inspect individual contact sites or microscopic details (6, 19). Here, the population of SACs reconstructed at EM resolution within the same retinal patch provides a perspective on both the global pattern and microscopic details combined.

## Results

### Ascending climbing dendrites of Off SACs

**Figure 1:**
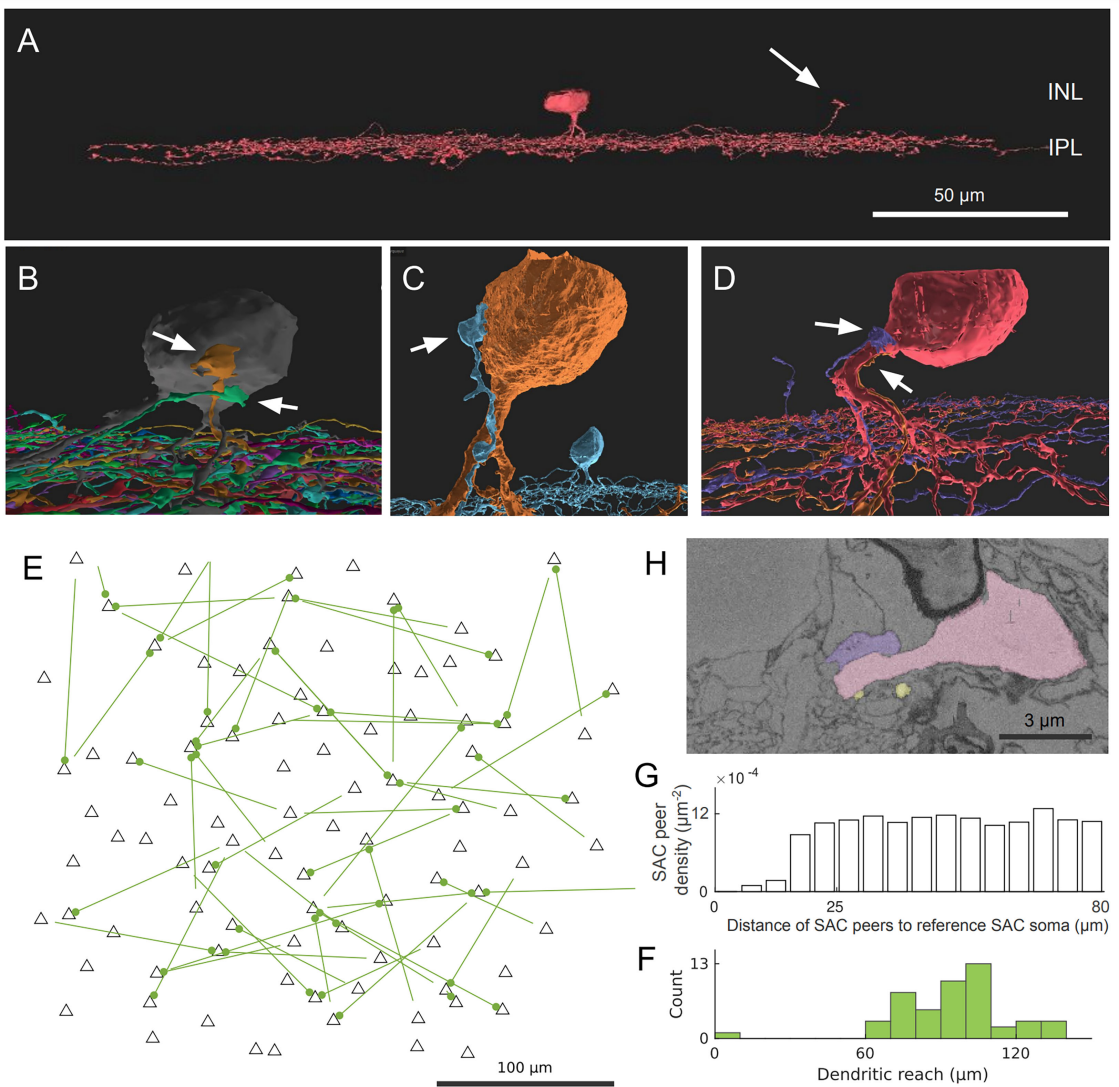
Off SAC contact pattern. **(A)** Tangential view of a 3D-reconstructed Off SAC. An ascending dendrite (arrow) veers off from the dendritic stratification and into the inner nuclear layer (INL). **(B–D)** In each of these 3D perspective views, attached to the soma or basal dendrite of an Off SAC, we see ascending dendrites (arrows) from other Off SACs like in **(A)**, usually climbing along the perisomatic dendrite. **(E)** Spatial distribution of the somatic origin and attachment points of these ascending dendrites in the retinal patch (flat-mount view). Each line represents the dendritic branch starting from the originating cell’s soma (the bare end of the line) and grasping onto the targeted cell’s perisomatic membrane (the bulged end of the line as a dot); black triangles are soma locations of all reconstructed Off SACs with soma inside the retinal patch. **(F)** Histogram showing the distribution of these ascending dendrites’ dendritic reach, defined as the planar distance from the ascending dendrite’s originating soma to the soma where it terminates. Minimum, quartiles, and maximum: 9, 81, 97, 104, and 137 μm. **(G)** The density recovery profile, for all Off SAC somas, regardless of whether any ascending dendrite contact or not, is defined as the average density of somas at given distances from any given soma (20). **(H)** A mini region of the plasma-membrane-stained retina sample, shown as a sectional electron micrograph near the locations pointed to in **(D)**, overlaid with the respective reconstructed cells’ colors matching panel **(D)**. Scale bars: 50μm **(A)**; 100μm **(E)**; 3μm **(H)**.

We found an interesting type of contact between Off SAC cells that involves the dendritic termination of one Off SAC and the soma and/or proximal dendrites of another Off SAC. These terminating dendrites veer towards the inner nuclear layer (INL) and, in many cases, travel almost parallel to the light axis (**Figure 1A**). This is unexpected because Off SAC dendrites normally stratify at a particular depth in the inner plexiform layer (IPL) and also terminate at that depth. The terminating dendrite often travels in contact with a proximal dendrite of the partner Off SAC, and if it reaches the partner cell’s soma, it typically spreads into a lump as it terminates (**Figures 1B–D**; **Supplementary Figure 2**). A majority of Off SAC cells (59 out of 96) in our dataset display this type of outbound and/or inbound contact with one or more other Off SAC cells. Within these 59 cells, 38 radiate as many as 3 contacts each (1.3 ± 0.6, mean ± s.d.) to other cells, and 39 somas receive as many as 4 (1.3 ± 0.6, mean ± s.d.) contact patches from other cells.

While no obvious pattern was seen (**Figure 1E**) in these perisomatically contacting Off-SAC to Off-SAC branches, we do see that the two cells in each contacting pair are rarely close to each other in terms of their somatic locations (**Figure 1F**). This can be attributed to the fact that in the flat-mount planar view, these contacts are often located near or at the most distal end of the dendrites. Of the 52 pairs of contacts we observed, the dendrite was reaching for the SAC soma nearest to the originating soma in only one case, while all other pairs were more than 60μm apart by soma distance (**Figure 1F**). In comparison, the density recovery profile (20) representing the distribution of all Off SAC somas had already reached a plateau at a distance of approximately 20-25μm (**Figure 1G**), indicating that the closest SAC neighbor of a SAC was almost always less than 20μm away.

### Direct contacts and short processes bridging On SAC somata

**Figure 2:**
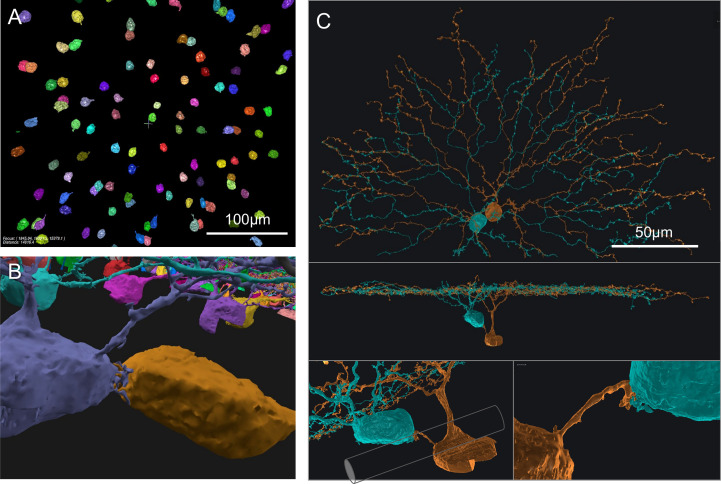
On SAC distribution and contacts. **(A)** Distribution of On SAC somas (colored objects are the 3D-reconstructed full or partial somas) in a flat-mount view of the retinal patch. **(B)** A pair of contacting On SACs form twigs at their soma contact (also in the background are somas of other On SACs, often incompletely reconstructed due to dataset boundaries). **(C)** A pair of On SACs that were next to each other in flat-mount view (top) did not have direct soma contact but were instead bridged by a short process between the somas (additional views in the middle and bottom panels). The two somas were separated by an axon bundle (illustrated by a cylindrical shape overlay within the bottom left panel) of retinal ganglion cells. Scale bars: 100μm **(A)**; 50μm **(C)**.

We also found interesting contacts at the somas of On SACs. Retinal neurons from a single-cell-type population, including SACs, were often considered to be more or less regularly spaced, forming a so-called mosaic arrangement, and rarely touch each other at the soma (8, 20, 21). However, in a prior study of SAC populations, Whitney et al. (19) reported a higher number of "close-neighbor pairs" in the ganglion cell layer (On SACs) than in the inner nuclear layer (Off SACs). Consistent with this observation, we found On SACs often paired up next to each other in the ganglion cell layer (**Figures 2A, B**). Unexpectedly, we noticed the pairs formed intertwined short twigs at the contact between the two somas (**Figure 2B**). In our specimen, 37 out of the 103 On SACs formed 19 adjoining pairs, with 14 pairs judged to have directly abutting somas (**Figure 2B** and **Supplementary Figure 1**) and the remaining 5 pairs having dedicated short branch(es) reaching between the two somas from within the ganglion cell layer (**Figure 2C**). We consider two cells as a pair only if one of these two preceding forms of contact is present, directly within the ganglion cell layer between the two 3D-reconstructed cells. All pairs have flat-mount center-to-center soma distances within 17μm, and those for directly abutting pairs are all within 13μm (9 ± 2, mean ± s.d.). For reference and comparison, our On (and Off) SAC population has soma diameters of ~9μm measured spherically, computed from soma volume as if each soma was a perfect sphere (soma volumes: 413 ± 30 μm^3^, On SACs; 389 ± 21, Off SACs; mean ± s.d. It should be noted that there is an uncorrected 7% linear shrinkage from the tissue preparation for EM imaging in all measurements, as detailed in the Methods section). The soma diameter of SACs measured under light microscopy was reported to be 10 (19, 22) to 11 μm (23). All pairs exhibit twigs intertwined to various degrees, with the least prominent form being short stubs protruding from the cell body, hugging, or protruding into the other cell body (**Supplementary Figure 1**).

Whitney et al. (19) argued that closer neighbor pairs were formed by cells displaced during development by fascicles of optic axons and retinal vasculature from their original mosaic-proper locations. However, we have seen an example pair of two On SAC somata straddling an optic nerve fascicle, and they still have a dedicated short process bridging them (**Figure 2C**). Optic nerve fascicles can be a hindrance to forming pairs of cells abutting each other and are therefore unlikely to be the cause of such formations. Another pair wrapped around a blood vessel, covering about 200 degrees of the blood vessel’s cross-sectional circumference. The blood vessel failed to cleanly separate the two somas, which remained touching hands (data not shown). Combined with the intertwined twig-like structures present in all pairs, these observations suggest that the formation of pairs may have functional or developmental significance. The occurrence of pairs was also reported in the rabbit in the ganglion cell layer (On SACs) and not in the inner nuclear layer (Off SACs) (24), and similar higher rates of occurrence in the ganglion cell layer can be observed from published figures and images for cat retina (Figure 4 in 21) and rabbit retina (25; Figure 7 in 26).

## Discussion

### Off SAC perisomatic contacts

Ray et al. (6) studied SAC development and reported that Off SACs establish dendrite-soma contacts during radial migration and assume transitory bi-laminar dendritic morphology that includes a soma-layer lamina with soma-layer SAC-SAC contacts upon completion of the migration. These soma-layer contacts, however, are mostly eliminated by day P3. It is possible that the dendrite-soma contacts we observed are remnants of these developmental processes. The retinal dataset we have is from a wild-type (C57BL/6) mouse of age P29 (10). On the other hand, the perisomatic contacts we observed were rarely (1 out of 52, **Figures 1F, E**) between two close-by neighboring Off starburst cells. These ascending dendrites are usually far away from their originating somata and are close to, or themselves are, the far terminating tip of the SAC dendrite carrying them. There are therefore usually several cell bodies of other starburst cells between the two cell bodies of the contacting pair of this kind. This is in contrast to the soma-layer contacts that Ray et al. (6) reported, which were exclusively between neighboring SACs.

SACs are particularly important for direction-selective information processing in the retina. Within individual starburst cells, different dendrites are known to function quite independently of each other in experiments examining light-evoked responses (17, 18, 27, 28). The occurrence of our ascending-dendrite contacts becomes quite rare if we compare against the total number of distal dendrites rather than the number of cells, i.e., if we were to regard these dendrites just like other (independently functioning) distal dendrites and if they relay and compute just the same kind of information a regular SAC distal dendrite relays. This rareness and perceived insignificant contribution can call for an argument that light-invoked responses are less likely to be the affected functional targets, except for perhaps long-range interactions across the retina or extremely local information where a single ascending dendrite should dominate all.

This known functional independence of individual dendrites pertains to light stimuli (with spatial details) but does not preclude potentially non-independent regulatory functions, for example, for developmental purposes. The fact that these contacts are on the cell bodies hints at a more cell-centric function than a local dendritic-centric function.

Not all Off SAC cells were observed to have this type of contact on them. However, with the extremely high coverage factor of starburst cells in the retina (exceeding 30; 29), just a small portion of these cells would already have the capacity to cover the entire retina.

### On SAC short bridging processes

In Ray et al. (6), similar to Off SAC dendrite-soma contacts, On SACs also made soma layer projections between days P0 and P3 that contacted neighboring SAC somata. The transcellular signaling protein Megf10 was found to promote the formation of the dendritic sublayer within the inner plexiform layer and the elimination of arbor projections in the soma layer by P3. The same protein additionally controlled the development of proper mosaic spacing of somas beyond P3 (6, 23). It is possible that the bridging processes we found were to help push neighboring somas apart before they eventually degenerate or retract, but such a possibility is remote given that the somata in these pairs we saw were all immediately adjacent to each other in the flat-mount projection view, constituting a gross violation of the frequently acknowledged near-soma dead zone (20, 30) or mosaic rule (25, 31). Abutting On SAC soma pairs were not specifically reported by Kay et al. (23) but were indeed visible and frequent in Figure S3 for wild-type mice, making our observations consistent with theirs.

Ray et al. (6) additionally observed single unbranched processes extending from the somata, ~180˚ away from the IPL. These 180˚ arbors were reported to be sometimes still present in P5 SACs and were considered to be fundamentally different from the tangentially projecting soma-layer neurites in the developing retina. The bridging processes in our P29 retina are also unbranched and in principle could also potentially be related to this second class of unusual processes.

With only morphological and patterning information available from this dataset, we can only speculate about the function or origin of these soma-layer contacts between close neighbors. Interestingly, a recent report documented that a fraction of On SACs fire (sodium-channel mediated) action potentials during cholinergic and glutamatergic retinal waves in the postnatal days. Specifically, only On SACs, and not Off SACs, were found to exhibit this spiking property, although the proportion of the firing subset decreases with age (32). We speculate that the shorter distance and the intertwined twigs in our On SAC pairs may be related to this spiking phenomenon by way of better electrical coupling between these cells, which are known to be mutually excitative in the postnatal days to generate the retinal waves (33). Non-synaptic cell-to-cell communication *via* nanotubules has been reported in various cell cultures (34, 35), and can form between cultured neurons (36, 37), and was recently reportedly found *in situ* between an astrocyte and a cortical neuron (38). However, it is unclear why the twigs between our On SAC cells would often need to be tortuous, entangled, and spatially clustered if they were nanotubules. These twigs at the contact surface or the end of short processes are perhaps more consistent with the morphologies of some of the synaptic invaginations in certain specialized forms of synapses (**Supplementary Figure 1**) (39, 40).

For mammalian retinal cells, there have been a few reports of perisomatic contact specializations or synapses. These include synapses from photoreceptor somata (41, 42), ribbon synapses from calbindin-positive rabbit ON cone bipolar cells (43), and somato-dendritic, somato-somatic, and dendro-somatic synapses from amacrine cells to amacrine cells, bipolar cells, and ganglion cells of non-specific types (44–47). Tyrosine hydroxylase positive (TH+) amacrine cells assemble perisomatic rings on multiple retinal amacrine cell types, including on SACs and characteristically on AII amacrine cells (48, 49). AII amacrine cells in turn form somatic synapses onto both sustained and transient Off-alpha ganglion cells (50). Both an Off-alpha and an Off-beta ganglion cell from a cat retina were found to receive amacrine cell synapses on their somata, which were presumed to be inhibitory (51). Mouse On-Off direction-selective ganglion cells have been identified to receive GABAergic somatic innervation from amacrine cells (52–54). A recently identified sparsely branched SB3 ganglion cell (55) and a bistratified GABAergic amacrine cell (56) in a rabbit retina received amacrine cell inputs on their somata. Lastly, it was recently demonstrated that TH+ cells in rat retinas have both excitatory and inhibitory synaptic receptor-expressing sites on their somatic surfaces (57). In our image volume, another form of perisomatic contact we have observed in a few cases was long-range traversing beaded single branches, making contact on somata of Off SACs and other amacrine cell types, possibly originating from TH+ cells. In the brain, perisomatic synapses are relatively well-known for the GABAergic network, especially the perisomatic clusters and rings (baskets) formed by basket cells (58).

Our reconstruction is known to be incomplete within the nuclear layers due to the dataset boundary and because the automated convolutional neural network algorithm that facilitated our reconstruction was not especially well-trained for the deep nuclear regions. The branches in both cases of the SAC subpopulations are thin and can be missed due to staining gaps or just proofreading oversights. For the On SACs, it is possible and likely that we missed some of the connecting branches, especially overpassing the data boundaries at the ganglion cell layer. For the Off SACs, it is also possible that we missed certain contacting patches if these dendrites reach well into the inner nuclear layer. However, the novel contacts we found are still abundant and are not unique cases of random mutations.

While it is not entirely surprising that electron microscopic reconstructions give finer views into the complex network of neuronal connections, we were still amazed by these novel contacts that had not been reported by prior studies. We believe the reasons why these were never reported before are twofold: first, in light microscopy, these cells need to be filled sparsely or differentially in order for these climbing dendrites and contacts to be seen, and staining efficiency and the signal-to-noise ratio become limiting factors in recognizing these contacts at the far-tip. Electron microscopy does not have these limitations but is typically done in tiny volumes. Second, neither light nor electron microscopy was traditionally volumetric, and these contacts only become apparent when a fully 3D visualization is employed, which is advantageous compared to single-section visualizations.

Due to historical reasons, this particular electron microscopy (EM) volume we used did not have intracellular staining, and we were unable to identify these nuclear-layer contacts as synaptic or otherwise (**Figure 1H**). Further studies of the subcellular structure and molecular identities are warranted for these special SAC-SAC contact sites.

## Methods

### EM image volume

The raw EM dataset was the e2198 volume from 10. Briefly, the dataset was from the retina of a P29 wild-type C57BL/6 mouse, and the tissue was fixed and specifically stained for plasma membranes. A 0.3 × 0.35 mm^2^ patch of retinal tissue was imaged by serial block-face electron microscopy from the ganglion cell layer to the inner nuclear layer and resulted in the e2198 EM volume with a spatial resolution of 16.5 × 16.5 × 23 nm/voxel. After examining the dimensions from previous two-photon calcium imaging microscopy of the same retinal tissue in the live state (10, 13), we found a tissue shrinkage of approximately 7% due to the preparation for EM imaging. Consistent with all previous publications using this EM volume, we chose to report all dimensions without correction for this shrinkage factor. Plasma membranes appear dark in the image volume, but intra-cellular membranes or organelles were not visible.

### Neuron reconstruction

Neurons were mostly reconstructed using the online citizen science game Eyewire.org, as reported in previous publications (11–13), and as part of more recent campaigns in the game. Additional efforts were also made to search for characteristic Off SAC patches and climbing dendrites on some somas where no incoming SAC contact had yet been observed; when such patches or dendrites were found, their locations were inserted into the Eyewire system as seeds for reconstruction. A small number of these found instances were back-traced to existing SAC reconstructions where the branches had previously been missed or mistaken for reconstruction errors due to their unusual course of extension. A number of them resulted in the reconstruction of full starburst cells that had not yet been reconstructed in the normal course of campaigns in the Eyewire game at the time.

Soma size computations were performed in the same manner as described in 13.

### Density recovery profile of Off SAC somas 

We computed the density recovery profile (20) in (**Figure 1G**) using all Off SAC somas as reference points, including those closer to the dataset boundary.

First, we computed all pairwise flat-mount planar distances between Off SAC somas and binned them into 5μm bins. Without normalization, this would result in a traditional histogram plot. We then normalized each bin count by dividing it by the area of the rings at the given planar distance from SAC somas in the dataset (respecting dataset boundaries, as seen below), thereby obtaining the density recovery profile. Each pairwise distance was counted twice due to the symmetric relationship between the two members of each pair.

For the inclusion of somas closer to the boundary, we did not use the method of correction factors (20), which uses mean effective sampling areas and relies on the assumption of a relatively uniform distribution of reference points, which would be entirely reasonable if the number of reference points was large enough. Instead, the concentric annuluses centered at each soma location were intersected with the bounding rectangle of the soma centers of these SACs, to produce the actual intersection areas which are used for the normalization described in the previous paragraph.

## Data availability statement

The original contributions presented in the study are publicly available. This data can be found here: https://museum.eyewire.org.

## Ethics statement

Ethical review and approval was not required for the animal study because existing published dataset.

## Author contributions

Eyewirers reconstructed neurons with supervision from MS, CD, DJ, and DB. SM observed the initial contact pattern and oversaw the overall data curation with help from MS. WS exported Eyewire data into the Neuroglancer software that facilitated searches for unreconstructed contacts. SM, MS, CD, DJ, and DB performed the search for unreconstructed contact patches on Off SAC somas. NT created an automated reconstruction of the somas at a lower resolution and extracted soma locations and sizes with proofreading by MM. SM performed the analyses and wrote the paper with feedback from NT and HS. WS ministered the online Eyewire Museum. CJ, WS, NK revamped much of the Eyewire infrastructure enabling transition of the game website from locally hosted servers to cloud service providers. CJ improved player reconstruction tools by adding client-side real-time marching cubes meshing and previewing functionality. AS oversaw addition of player-added utilities and scripts, and other game features implemented by CJ, WS, NK, such as live reconstruction activity overviews. All authors contributed to the article and approved the submitted version.

## Supplementary material

Supplementary Datasheet 1: Supplementary Figures This file includes additional examples and close-up views of the contact sites in both EM sections and 3D views.

Supplementary Datasheet 2: Supplementary Notes This file includes a list of individual Eyewirers who contributed to the reconstruction of the starburst cells in this report.
