# Supplementary material for: Special nuclear layer contacts between starburst amacrine cells in the mouse retina
Source: Front Ophthalmol (Lausanne). 2023 Mar 24;3:1129463. doi: 10.3389/fopht.2023.1129463 (PMC11182129; doi:10.3389/fopht.2023.1129463)
Supplement: Supplementary Datasheet 1: Supplementary Figures — This file includes additional examples and close-up views of the contact sites in both EM sections and 3D views. [file DataSheet_1.pdf]

Supplementary Figures

Supplementary Figure 1

Additional examples of contacting On SAC somas

Top row: Electron microscopic sectional views of the contacting surface in different pairs. The contacting somas (asterisks) form protrusions and twigs, commonly at the the contacting surface boundary - intertwined (paired arrows) or hugging the partner soma (singular arrow), and sometimes in the middle of the contacting surface (arrowheads), with the last example being an "entangled ball" formed by the intertwined twigs right in the middle of a soma-soma contacting surface (paired arrowheads).

Bottom row: 3D perspective views showing the “hugging” type of protrusions at the boundaries of the main contacting surfaces. The yellow-brown colored cell in the third example is shown from two different angles, where the contacting surface is shown obliquely facing our left side and surrounded by a few twigs in the bottom view (purple cell hidden).

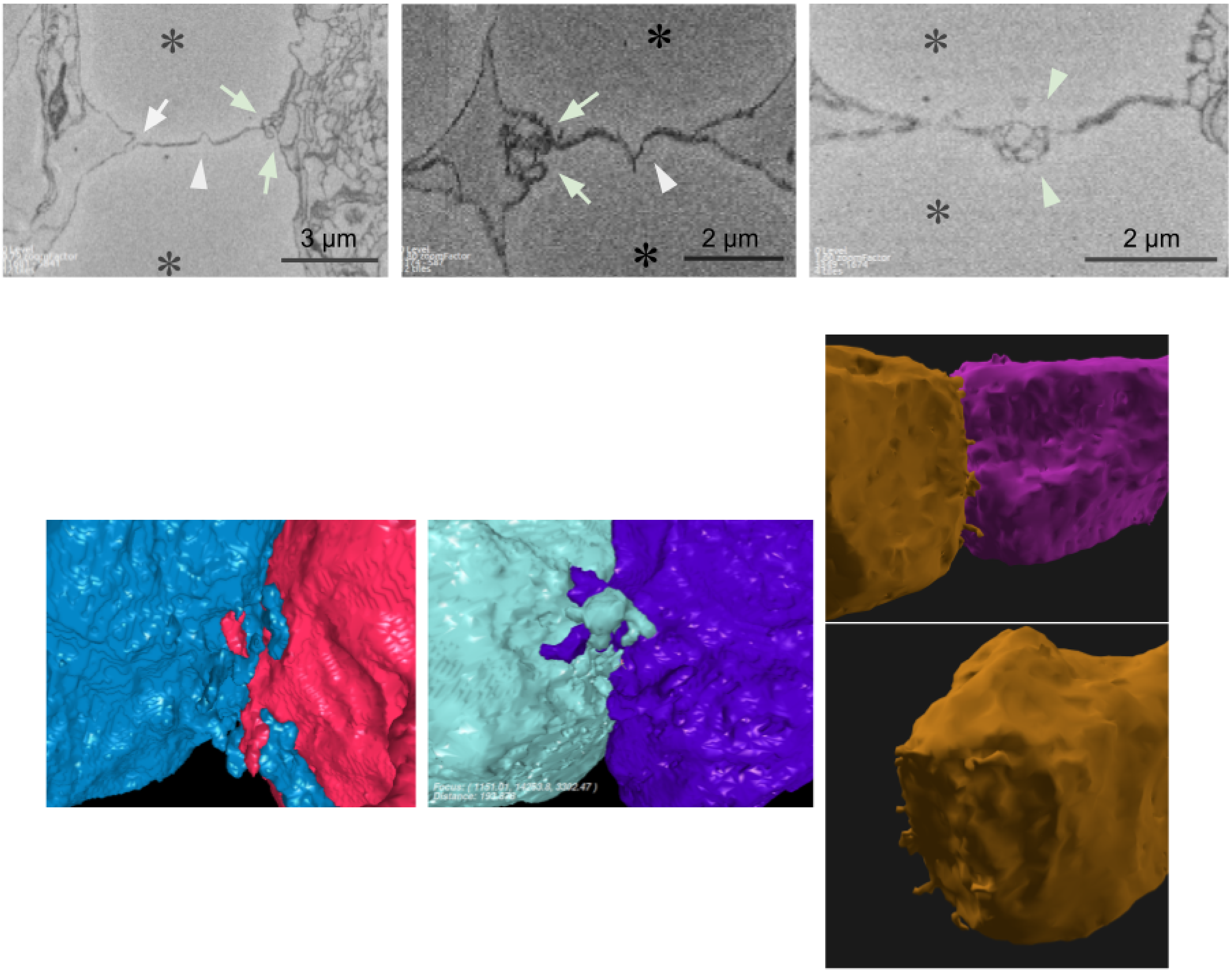

Supplementary Figure 2

Additional examples of ascending dendrite contacts on Off SAC somas (orthogonal 2D sectional views of the electron microscopic volume and color-matched 3D views from various directions)

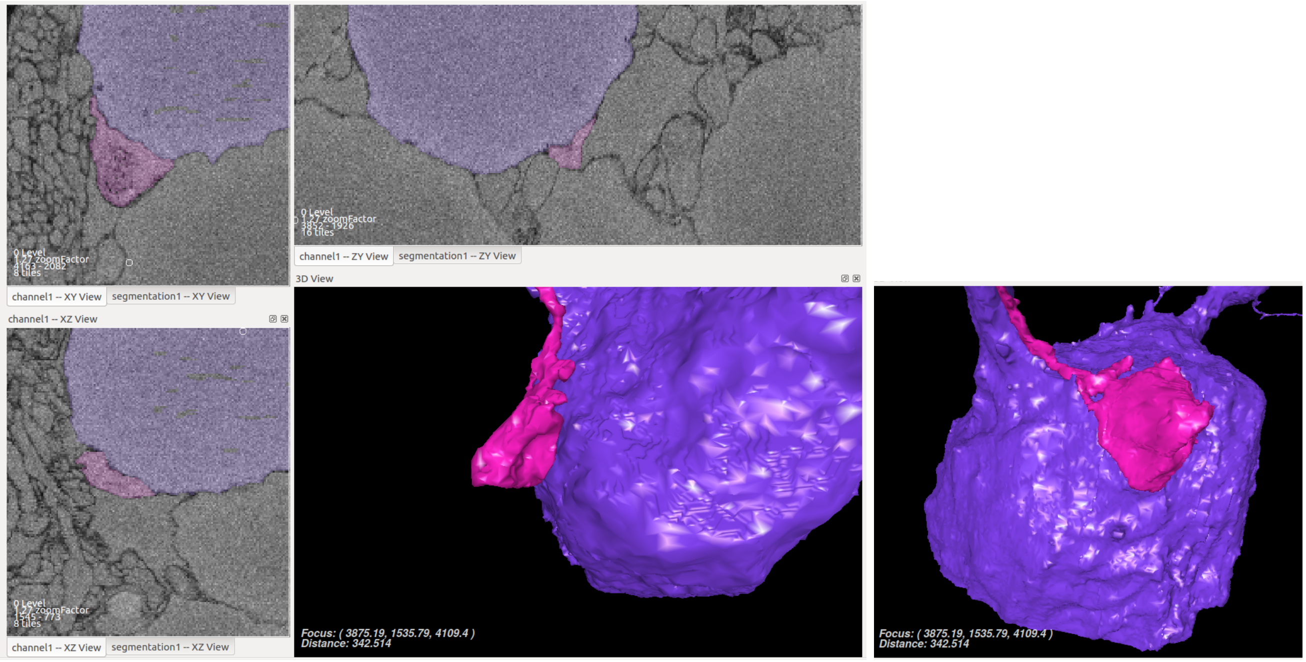

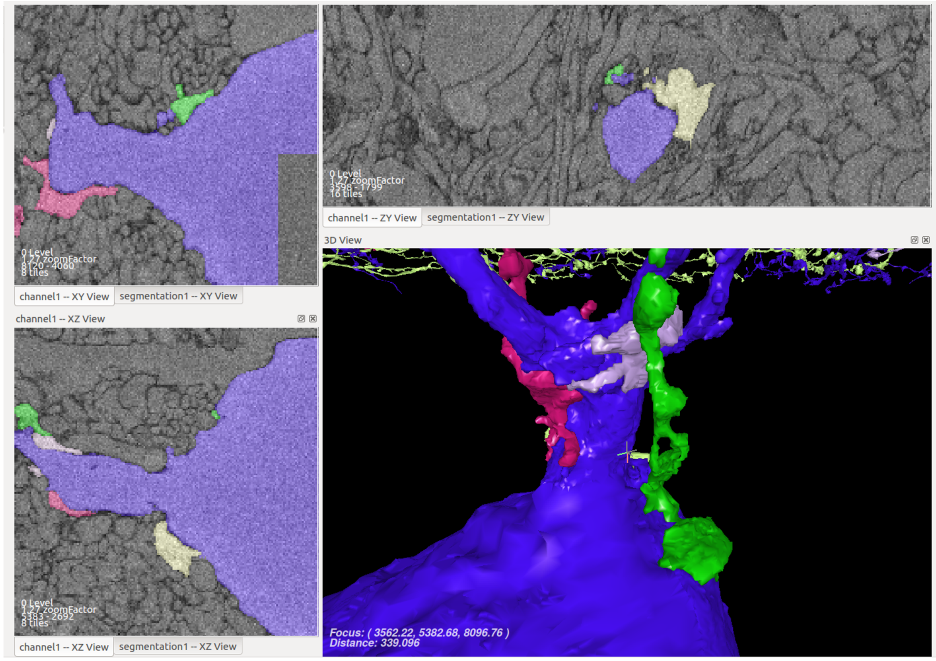

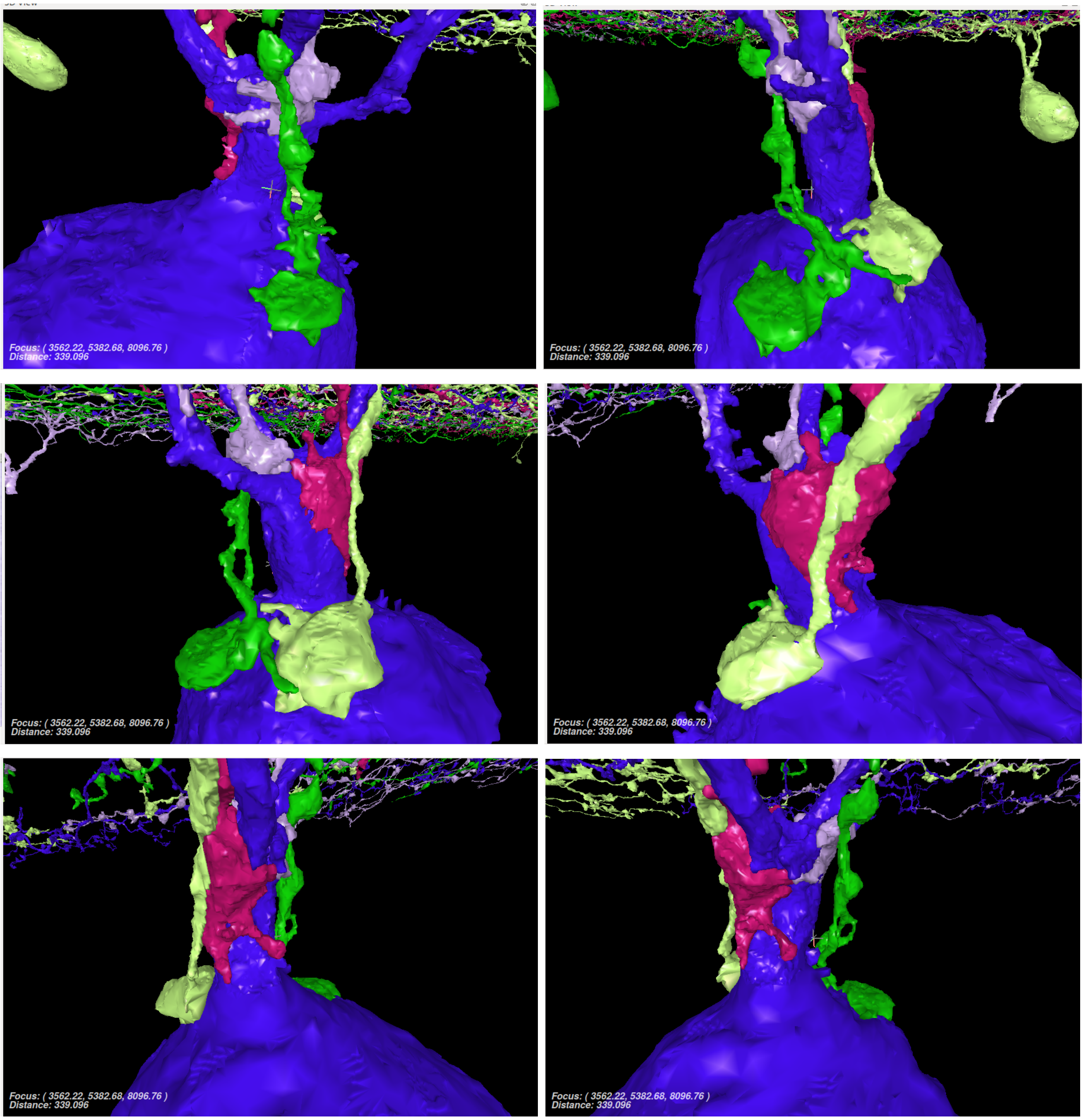
Focus: (3562.22, 5382.68, 8096.76)
Distance: 339.096
Focus: (3562.22, 5382.68, 8096.76)
Distance: 339.096
Focus: (3562.22, 5382.68, 8096.76)
Distance: 339.096
Focus: (3562.22, 5382.68, 8096.76)
Distance: 339.096
Focus: (3562.22, 5382.68, 8096.76)
Distance: 339.096
Focus: (3562.22, 5382.68, 8096.76)
Distance: 339.096
